# Is All Weight Loss Equal Following Sleeve Gastrectomy? Defining Body Composition and Anthropometric Thresholds for Hyperglycemia Remission in Women from a Prospective Cohort Study

**DOI:** 10.1007/s11695-026-08570-z

**Published:** 2026-03-11

**Authors:** Louise Becroft, Priya Sumithran, Paul Burton, Wendy Brown

**Affiliations:** 1 Nutrition and Dietetics Department, Alfred Health, Melbourne, Australia; 2 Department of Surgery, Monash University, Melbourne, Australia; 3 Department of Surgery, Monash University, Melbourne, Australia; 4 Department of Endocrinology and Diabetes, Alfred Health, Melbourne, Australia; 5 Oesophago-Gastric and Bariatric Unit, Alfred Health, Melbourne, Australia

**Keywords:** sleeve gastrectomy, hyperglycemia remission, body composition, central adiposity

## Abstract

**Introduction:**

Adiposity magnitude and distribution strongly influence the risk of prediabetes and type 2 diabetes (T2D). While metabolic bariatric surgery provides durable weight loss and metabolic improvement, optimal anthropometric and body composition targets for hyperglycemia remission remain undefined.

**Methods:**

We conducted a prospective cohort study of women undergoing sleeve gastrectomy (SG) in Melbourne, Australia (2019–2022). Anthropometric, biochemical, and dual-energy X-ray absorptiometry (DXA) derived body composition data were collected at baseline and 12 months post-surgery. The primary outcome was change in body composition. Secondary outcomes included percentage total body weight loss (%TBWL), fat-mass loss, and central adiposity thresholds associated with hyperglycemia remission, defined as HbA1c < 6.0% or fasting glucose < 6.0 mmol/L without medications.

**Results:**

Of 50 patients recruited, 39 completed the study. Marked reductions in fat mass and central adiposity were observed. All seven participants with T2D achieved hyperglycemia remission; five discontinued hypoglycemic therapy. Among those with prediabetes/T2D (*n* = 14), 93% achieved hyperglycemia remission. ROC analyses identified candidate thresholds associated with remission, including ≥ 21.9% TBWL (AUC 0.85), ≥ 75.8% fat-mass contribution to weight loss (AUC 0.85), postoperative waist circumference ≤ 107 cm (AUC 0.93), and waist-to-height ratio ≤ 0.72 (AUC 0.85). Logistic regression combining %TBWL and WHtR yielded an AUC of 1.00; however, this likely reflects overfitting given the very small remission subgroup.

**Conclusion:**

Total weight loss and reductions in central adiposity were strongly associated with hyperglycemia remission after SG in women. Achieving 20–25% TBWL, ≥ 75% fat-mass contribution to weight loss, and postoperative waist circumference < 107 cm may represent candidate treatment targets, but require validation in larger independent cohorts.

## Key Points

Reductions in central adiposity were more strongly associated with hyperglycemia remission than BMI-based measures alone.

Hyperglycemia remission was common, with 93% of participants with prediabetes or T2D achieving this.

Postoperative thresholds of ≥ 21.9% TBWL, ≥ 75.8% fat mass loss, waist circumference ≤ 107 cm and waist-to-height ratio ≤ 0.72 were strongly associated with hyperglycemia remission.

These exploratory thresholds may help guide postoperative monitoring but require validation in larger, independent cohorts.

## Introduction

Obesity affects over 880 million adults globally and its prevalence continues to rise, with projections indicating continued growth across all regions [1, 2]. It is a heterogeneous condition characterized by wide variation in fat quantity and distribution [3]. Central adiposity is particularly harmful, being strongly associated with systemic inflammation, insulin resistance, type 2 diabetes (T2D), cardiovascular disease, hepatic steatosis, and several cancers [4]. Obesity-related metabolic dysfunction contributes substantially to global disease burden, with T2D affecting over 500 million people worldwide [5].

Obesity is most commonly defined using Body Mass Index (BMI), despite its inability to distinguish between fat and lean mass or capture adipose distribution [3]. Measures of central adiposity, such as waist circumference (WC) and waist-to-height ratio (WHtR), are more predictive of metabolic risk than BMI [6, 7]. Increasing evidence supports the use of body composition-based measures as more accurate and clinically meaningful predictors of health outcomes [8, 9].

Metabolic bariatric surgery (MBS) is the most effective and durable treatment for obesity, inducing substantial weight loss and metabolic health improvement [10]; with sleeve gastrectomy (SG) now being the most widely performed procedure [11]. T2D remission after MBS is typically defined by achieving HbA1c < 6.5% without medication for at least 3 months [12]. Landmark trials such as STAMPEDE and the SOS study [13, 14] reported high T2D remission rates but did not define anthropometric or body composition thresholds that might underpin metabolic improvement, limiting translation into practical treatment targets.

Most MBS recipients globally are female, yet there is a paucity of data describing body composition changes after SG in this cohort [11]. Given women are more likely to experience age-related sarcopenia than men [15], understanding the composition of weight loss after SG is clinically important.

The primary aim of this study was to measure body composition and anthropometric changes after SG in females. Secondary aims were to identify the minimum percentage total body weight loss (%TBWL) associated with hyperglycemia remission and to determine fat mass reduction and central adiposity thresholds that best predict hyperglycemia remission following surgery. These thresholds were derived using ROC analyses and logistic regression models. As weight loss and metabolic improvements after SG typically reach their maximal effect within the first 12 months [16, 17], this time point represents the most clinically relevant window for assessing body composition and glycaemic outcomes.

## Methods

### Study Design and Participants

**Fig. 1:**
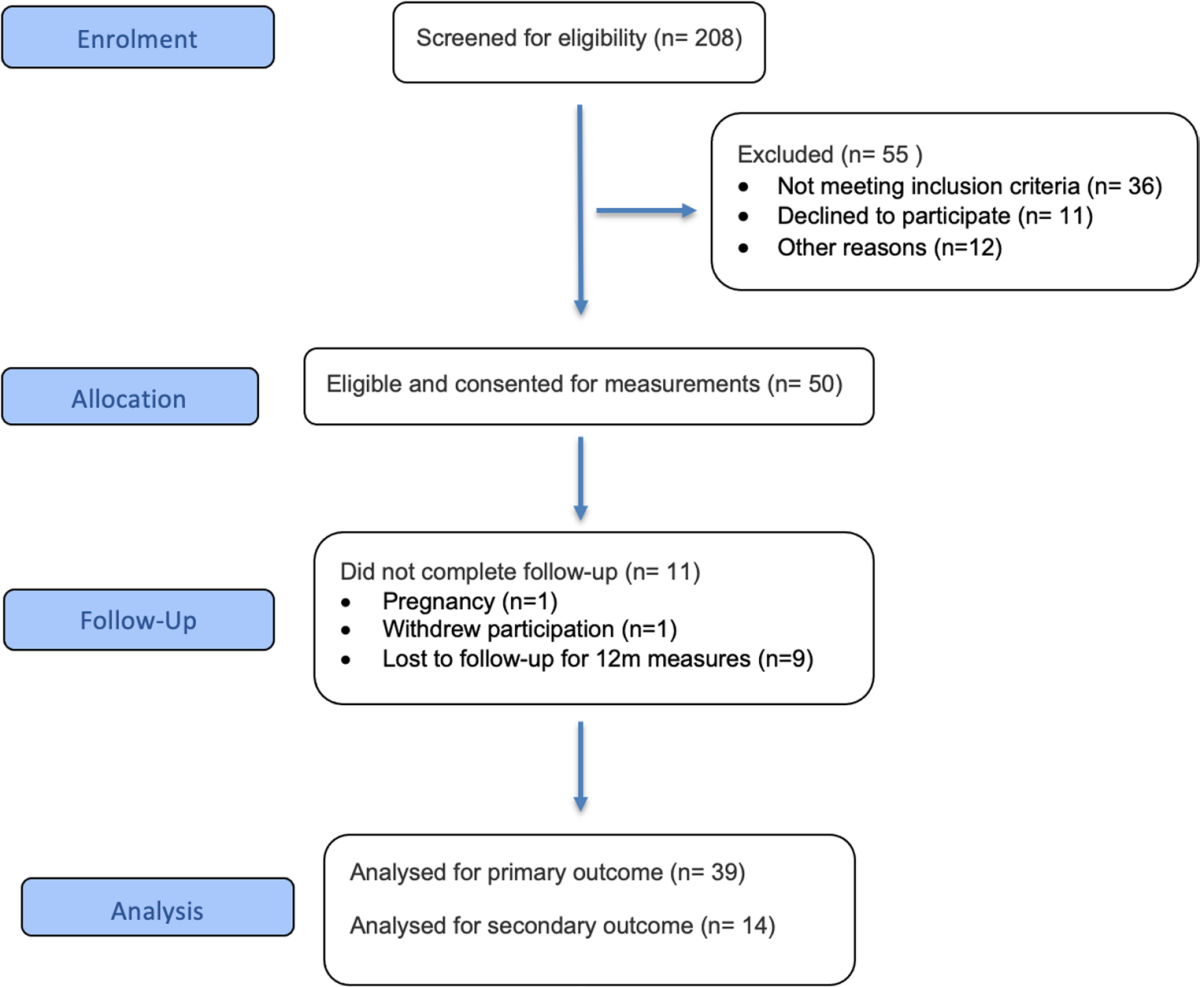
CONSORT 2025 flow diagram of participant enrolment

We conducted a prospective cohort study of female patients undergoing primary sleeve gastrectomy between January 2019 and December 2022, at public and private hospitals in Melbourne, Australia. Inclusion criteria were female sex, age ≥ 18 years, undergoing primary sleeve gastrectomy, and ability to provide informed consent. Exclusion criteria were previous bariatric surgery, pregnancy, inability to undergo DXA scanning. Analyses were performed using complete cases only, no imputation was undertaken. A total of 39 participants with complete 12-month anthropometric, biochemical and body composition datasets were included in the final analysis. Reasons for missing data and loss to follow-up are detailed in the CONSORT flow diagram Fig. 1, prepared in accordance with the CONSORT 2025 reporting guidelines [18]. Although CONSORT is designed for randomized trials, we used its flow-diagram format to report participant recruitment and retention, consistent with recommendations for clear reporting when STROBE diagrams are not mandated.

### Procedures

Ethical approval was obtained from local hospital ethics committees, and the trial was retrospectively registered on the clinical trials registry. All procedures complied with the 1964 Helsinki declaration.

Anthropometric, biochemical, and body composition data were collected at baseline (within 1 month pre-surgery) and again at one year (12–15 months) post-operatively in a fasted state.

Weight was recorded to the nearest 0.1 kg. Height and WC were measured to the nearest 0.1 cm using standardized protocols. Body composition was assessed using dual-energy X-ray absorptiometry (DXA; Lunar iDXA). Due to scanner size constraints, half-body DXA scans were performed, and whole-body fat and lean mass were estimated using the manufacturer’s validated half-scan reconstruction algorithms. Although these algorithms are widely used, they may introduce some measurement error, which should be considered when interpreting the results.

Biochemical analyses included HbA1c and fasting plasma glucose. T2D was defined as HbA1c ≥ 6.5% and/or fasting glucose ≥ 7.0mmol/L [19], while prediabetes was defined as HbA1c as ≥ 6.0% and fasting glucose as ≥ 6.1mmol/L using Australian diagnostic criteria [20] at baseline. Diabetes medication use was recorded at baseline and 12 months. T2D duration was not systematically captured. In this manuscript, ‘hyperglycemia remission’ is used as the primary term, with ‘normoglycemia’ referring to the operational definition of remission (HbA1c < 6.0% or fasting glucose < 6.0 mmol/L without medications). This definition differs from the 2021 international consensus definition of diabetes remission (HbA1c < 6.5% for at least 3 months without glucose-lowering therapy), and this discrepancy may limit direct comparability with studies using the consensus criteria.

The primary outcome was change in body composition, defined as changes in total fat mass and lean mass at 12 months postoperatively. Secondary outcome included %TBWL associated with hyperglycemia remission and thresholds of fat mass and central adiposity (WC, WHtR) that best predicted hyperglycemia remission.

### Sample Size

Using G*Power 3.1 [21] a sample of 40 participants was required to detect a 2 kg difference in fat or lean mass with a significance level of *p* < 0.05 and statistical power (1-β) of 80%. Allowing for attrition, a target of 48 participants was set.

### Statistical Analysis

Analyses were performed using IBM SPSS Statistics (version 30). Descriptive statistics summarized baseline and outcome measures. Between-group differences were assessed using t-tests or Mann-Whitney U tests. ROC analyses estimated optimal thresholds, 95% confidence intervals for all AUC estimates were calculated using the nonparametric method. Pearson correlations assessed associations. No correction for multiple comparisons was applied, and all p-values should therefore be interpreted as exploratory.

Logistic regression analyses were primarily conducted using univariable models, with a single predictor entered per model, due to the limited number of remission events. Multivariable logistic regression models including two predictors were additionally constructed to evaluate whether combinations of key anthropometric and body composition variables improved prediction of hyperglycemia remission. Multicollinearity was assessed using variance inflation factors (VIFs).

Model performance was evaluated using the area under the receiver operating characteristic curve (AUC). Given the small number of outcome events, the regression models are susceptible to instability and potential overfitting. Instances of perfect separation observed in the models should therefore be interpreted as overfitting arising from the limited sample size and few remission events. Internal validation was not performed and is acknowledged as a limitation. All analyses were conducted using complete cases only.

## Results

### Study Population

Between 2019 and 2022, 50 female patients underwent SG, of whom 39 had complete anthropometric, biochemical and body composition data for analysis (Fig. 1). Recruitment and follow-up were affected by COVID-19 related service disruptions at our site.

At baseline, 14 participants (36%) had prediabetes or T2D. There were no significant differences between the prediabetes/T2D cohort and those without for any baseline anthropometric measures (Table 1).

**Table 1:** Baseline demographic and anthropometric characteristics

	Prediabetes + T2D (*n* = 14)	Normoglycemia (*n* = 25)	*p*-value
Age, years	46.7 (12.2)	41.4 (11.5)	0.38
BMI, kg/m^2^	47.6 (6.7)	45.5 (6.4)	0.66
Weight, kg	125 (23.9)	123.1 (17.0)	0.95
WC, cm	131.7 (18.8)	129.7 (15.4)	0.83
WHtR	0.82 (0.1)	0.79 (0.1)	0.76
% body fat	44.1 (11.8)	44.8 (6.7)	0.97
% lean mass	42.1 (7.4)	43.2 (3.4)	0.62

Data are mean (SD). *P*-values are for comparison between cohorts with and without prediabetes/T2D using T-tests and Mann-Whitney U tests. *BMI*, body mass index; *WC*, waist circumference; *WHtR*, waist to height ratio; *T2D*, type 2 diabetes

In total, seven participants (18%) met diagnostic criteria for T2D at baseline (six by HbA1c ≥ 6.5% and one by fasting glucose ≥ 7.0 mmol/L); a further seven had prediabetes (HbA1c 6.0-6.4% or fasting glucose 6.1–6.9 mmol/L). Of those with T2D, five were on oral hypoglycemic agents pre-operatively. All seven with T2D achieved hyperglycemia remission (HbA1c < 6.0) and all discontinued medications after surgery. Among those with prediabetes, six of seven (86%) achieved hyperglycemia remission.

There were no significant baseline differences between glycemic groups. At 12 months, reductions in %TBWL, fat and lean mass were similar, while waist circumference reduction was significantly greater in those with prediabetes/T2D (Table 2).

**Table 2:** Anthropometric changes from baseline at 12 months postoperatively

	Prediabetes + T2D (*n* = 14)	Normoglycemia (*n* = 25)	*p*-value
% total body weight loss	21.1 (11.4)	24.3 (10.9)	0.41
% fat mass loss	68.1 (28.6)	78.4 (13.9)	0.24
% lean mass loss	26.4 (25.7)	19.8 (17.7)	0.43
WC reduction, cm	31.5 (11.1)	20.8 (13.3)	0.013
WHtR reduction	0.15 (0.1)	0.12 (0.08)	0.36

Data are mean (SD). *P*-values are for comparison between cohorts with and without prediabetes/T2D using Mann-Whitney U tests. *WC*, waist circumference; *WHtR*, waist to height ratio; *T2D*, type 2 diabetes

### HbA1c Targets

Receiver operating characteristic (ROC) analysis suggests optimal thresholds of 21.9% TBWL (AUC 0.85) and ≥ 75.8% of total weight loss attributable to fat mass (AUC 0.85) were able to discriminate hyperglycemia remission (HbA1c < 6.0%) following surgery for participants with prediabetes/T2D (Table 3).

T2D/prediabetes participants achieving hyperglycemia remission (*n* = 13) had a greater WC reduction (31.5 ± 11.1 cm) compared to non-responders (0.1 ± 10.7 cm; *p* = 0.013). ROC analysis (AUC 0.93) suggested a post-treatment waist circumference ≤ 107.0 cm as the optimal threshold for predicting glycemic normalization (*r* = 0.69, *p* = 0.009) in those with prediabetes/T2D (Table 3).

**Table 3:** Predictors of hyperglycemia remission (HbA1c <6.0%)

	Optimal threshold	Correlation r	*p*-value	AUC (95% CI)
% Total body weight loss	21.9	0.46	0.11	0.85 (0.54-1.00)
% Fat Mass Loss	75.8	0.12	0.75	0.85 (0.54-1.00)
WC, cm	≤107.0	0.69	0.009*	0.93 (0.74-1.00)
WC reduction, cm	31.5	0.73	0.013*	0.83 (0.49-1.00)
WHtR	≤0.72	0.18	0.27	0.85 (0.54-1.00)

*WC*, waist circumference; *WHtR*, waist to height ratio; *AUC*, area under the curve. *denotes significance

An optimal WHtR threshold of ≤ 0.72 was identified as a predictor of hyperglycemia remission postoperatively for those with prediabetes/T2D, with an area under the curve (AUC) of 0.85, suggesting strong discriminative ability.

Among patients with prediabetes/T2D, logistic regression identified %TBWL and post-operative waist-to-height ratio (WHtR) as the strongest combined predictors of achieving glycemic normalization (HbA1c < 6.0), the model demonstrated perfect classification (AUC = 1.00). Achieving 25.6% total weight loss and reducing WHtR to < 0.69 was associated with the highest likelihood of achieving glycemic normalization in this cohort.

### Fasting Plasma Glucose Targets

ROC analysis identified the optimal cut-off was 75.8% (AUC 1.00) among responders, this threshold perfectly discriminated responders from non-responders (*p* = 0.008). Correlation between percentage fat mass loss and normoglycemia was strong (*r* = 0.75; *p* = 0.012; Table 4).

**Table 4:** Predictors of hyperglycemia remission (glucose < 6.0mmol/L)

Predictor	Optimal threshold	Correlation (*r*)	*p*-value	AUC (95% CI)
% TBWL	13.5%	0.44	0.17	0.73 (0.28-1.00)
% Fat Mass contribution to TBWL	75.8%	0.75	0.012*	1.00 (1.00–1.00)
WC, cm	≤ 98.0	0.58	0.031*	0.77 (0.36-1.00)
WC reduction, cm	28	0.29	0.32	0.68 (0.18-1.00)
WHtR	≤ 0.662	0.29	0.324	0.735 (0.29-1.00)

*TBWL*, total body weight loss; *WC*, waist circumference; *WHtR*, waist to height ratio; *AUC*, area under the curve. *denotes significance

ROC analysis identified for those with prediabetes/T2D a post-surgery waist circumference ≤ 98.0 cm as the optimal threshold for normalization of fasting glucose (*r* = 0.58; *p* = 0.031). The correlation between waist circumference reduction and hyperglycemia remission status was weak and not statistically significant (*r* = 0.29; *p* = 0.32), Table 4. As expected, waist circumference thresholds differed according to the glycemic endpoint assessed, with a higher threshold predicting HbA1c-defined remission and a lower threshold predicting fasting glucose normalization.

WHtR was associated with moderate discrimination of glycemic normalization (AUC 0.735), with a threshold of ≤ 0.662. However, correlation between post-treatment WHtR and glucose levels was weak and non-significant (*r* = 0.285; *p* = 0.32). Logistic regression combining WHtR and the proportion of total weight loss attributable to fat mass resulted in perfect separation. This pattern is characteristic of overfitting and reflects the very small number of remission events rather than true predictive certainty.

## Discussion

In this prospective cohort study of women undergoing primary sleeve gastrectomy, we identified body composition and anthropometric thresholds strongly associated with hyperglycemia remission. Our findings emphasize that beyond total weight loss, reductions in fat mass, specifically reduction in central adiposity, are strongly associated with metabolic outcomes after MBS.

Body composition changes observed in this study, with fat mass loss ranging from 68 to 78% and lean mass loss ranging from 19 to 26% of TBWL, are consistent with previously reported outcomes after MBS. Most prior investigations have focused on Roux-en-Y gastric bypass in mixed gender cohorts [22–25], whereas findings specific to sleeve gastrectomy show greater variability in fat mass reduction [23, 26]. Maïmoun et al. found 32% total fat mass loss at 12 months in a small (*n* = 83) predominantly female cohort undergoing sleeve gastrectomy [27], while Diedisheim et al. investigated body composition changes at 12 months post sleeve gastrectomy in a predominantly female cohort, finding reductions in fat mass in those with and without T2D of 28% vs. 37% respectively [26]. Gender differences in body composition further contribute to this heterogeneity, with women exhibiting higher total body fat and more subcutaneous fat distribution than men both before and after surgery [28]. Findings presented in previous studies with a greater proportion of female subjects, such as Carey et al. [24], report up to 75% fat mass reduction, aligning closely with the present results.

### Interpretation in the Context of Existing Evidence

Previous landmark trials such as STAMPEDE and SOS established MBS as the most effective intervention for inducing T2D remission, yet primarily defined success using glycemic indices without reference to specific anthropometric or body composition thresholds [13, 14]. In the STAMPEDE trial, remission (HbA1c < 6.0% without medication) occurred in 37% of sleeve gastrectomy patients at 12 months postoperatively, compared with 12% in intensive medical therapy and a reported weight loss of 24.7% in the surgical group [29]. Similarly, the SOS study reported 72% T2D remission at 2 years after surgery, accompanied by a 21.3% TBWL [14]. By contrast, in our study, all seven patients with baseline T2D achieved remission at one year after surgery, and 93% of those with prediabetes or T2D achieved glycemic normalization (both HbA1c < 6.0% and fasting glucose < 6.0mmol/L) at one year after surgery. These remission rates appear numerically higher than those reported in STAMPEDE and SOS; however, such comparisons should be interpreted cautiously given the small sample size in the present study and the differing remission definitions used across studies. These remission rates are comparable to those reported in larger trials.

Importantly, we observed that ≥ 75% of total weight loss attributable to fat mass was associated with normalization of HbA1c and fasting glucose, highlighting the importance of the composition of weight loss, not merely its magnitude, for metabolic improvements. This is consistent with mechanistic studies suggesting that ectopic fat reduction in the liver, pancreas, and visceral depots improves β-cell function and insulin sensitivity, thereby facilitating durable glycemic improvements [30, 31]. Conversely, disproportionate lean mass loss may blunt metabolic benefits, underscoring the need for post-operative strategies that protect muscle mass through adequate protein intake and physical activity [32, 33].

Our data suggest that remission of hyperglycemia is achievable at total weight loss levels relatively modest for MBS (21% TBWL) if reductions in central adiposity accompany this change. The strong discriminatory value of waist circumference ≤ 107 cm and WHtR ≤ 0.72 suggests these values may function as exploratory candidate thresholds, rather than definitive clinical cut-offs, and supports the growing recognition of abdominal obesity as a key driver of insulin resistance and metabolic dysfunction [9, 34]. Notably, the waist circumference thresholds differed between HbA1c and fasting glucose defined remission, reflecting the use of distinctly different glycemic endpoints. The wide confidence intervals around several AUC estimates reflect the small number of remission events and indicate substantial statistical uncertainty, reinforcing that these thresholds should be interpreted as exploratory. As the study was powered only to detect changes in body composition, all subgroup analyses, including ROC-derived thresholds and logistic regression models, should be considered exploratory and hypothesis-generating rather than confirmatory. The small number of participants with prediabetes or T2D (*n* = 14) substantially limits statistical power for subgroup and predictive analyses. Accordingly, the apparent perfect discrimination observed when combining %TBWL and WHtR should be interpreted as hypothesis-generating rather than confirmatory, and requires validation in larger, independent cohorts.

### Clinical Implications

The thresholds identified in this study may have potential translational relevance and should be considered exploratory thresholds rather than prescriptive clinical cut-offs. First, they provide tangible treatment targets, enabling counselling framed around specific goals such as ≥ 20–25% total weight loss, waist circumference < 107 cm, or fat mass accounting for ≥ 75% of total weight loss. Such metrics more directly reflect changes in central adiposity than percentage excess weight loss or BMI-based categories, which provide weaker links to metabolic outcomes [3, 35]. Although the present findings were derived from measures obtained via DXA, routine use may be limited by cost, accessibility and feasibility of repeated measurements. Alternative modalities such as bioimpedance analysis, or where available, ultrasound and computed tomography, may provide practical approaches for longitudinal monitoring of body composition in clinical settings, however their application is also limited by cost, radiation exposure (CT) and accessibility.

Second, incorporation of simple central adiposity measures (WC, WHtR) into routine follow-up could refine postoperative monitoring, allowing early identification of patients at risk of persistent hyperglycemia despite substantial weight loss. Unlike advanced body composition assessment methods, that are often expensive and inaccessible in resource-limited settings, WC and WHtR offer practical advantages due to their affordability, ease of implementation and minimal training requirements [6, 7]. This is particularly relevant given the global epidemiological shift, where obesity is no longer being confined to high income countries, but becoming a pressing health challenge in low and middle income nations [1, 2, 36]. Adoption of these measures may offer robust, accessible and scalable monitoring tools for obesity management, supporting equitable healthcare delivery across diverse populations.

Finally, if confirmed in larger independent cohorts, these thresholds may inform future trial outcome measures and guideline development, offering a foundation for consensus on post-surgical treatment targets for normalization of glycaemia.

### Strengths and Limitations

A strength of this study is the use of DXA-derived body composition, the recommended method for patients with obesity [37]. Available techniques, such as anthropometry, bioelectrical impedance, DXA, CT and MRI each carry advantages and limitations including cost, accessibility, radiation exposure or reduced accuracy in individuals with high adiposity. DXA can offer accurate, reproducible estimates of fat and lean mass compartments with relatively low radiation burden. By mitigating the limitations of other approaches, DXA enhances the reliability and validity of our findings in this population. However, the use of half-body DXA acquisition represents a specific limitation, as reconstruction algorithms, although validated, may introduce additional measurement error, particularly in individuals with high adiposity.

The focus on a female cohort undergoing sleeve gastrectomy, reflects the real-world predominance of female patients undergoing the most popular MBS. The use of multiple analytic methods (ROC, correlations, logistic regression) increases confidence in the observed associations reported in this study.

Limitations must be acknowledged. The small sample size reduces statistical power and increases susceptibility to type I and type II error. Given the number of statistical tests performed, no correction for multiple comparisons was applied, which increases the risk of type I error and indicates that these findings should be interpreted as exploratory. The perfect separation observed in logistic regression reflects overfitting driven by the small sample size and limited number of remission events, rather than true predictive certainty. These findings need to be confirmed in an independent cohort. T2D duration, which is known to influence remission probability [38, 39], was not systematically collected. Because shorter diabetes duration is strongly associated with higher remission rates after MBS, the absence of duration data in our cohort is a major limitation and limits comparability with other trials. Large registry-based data from Sweden demonstrate a strong inverse association between diabetes duration and remission, with remission rates exceeding 70% in those with < 2 years’ duration but falling below 20% after ≥ 10 years [38]. Similarly, long-term follow-up studies report markedly higher and more durable remission among individuals with shorter preoperative diabetes duration [39], underscoring its importance as a prognostic factor.

Furthermore, the exclusive inclusion of women limits generalizability to males, who may exhibit different fat distribution patterns and metabolic responses. Men typically have greater visceral adiposity and may experience different patterns of fat loss and metabolic improvement after MBS; therefore, excluding male participants may limit the applicability of these thresholds to populations with sex-specific differences in adipose distribution and glycemic response. The limited number of remission events reduced model stability and increased the risk of overfitting, and internal validation was not performed; therefore, regression-derived thresholds should be interpreted cautiously.

### Future Directions

Future research should aim to validate these anthropometric and body composition thresholds in larger, prospective, and sex-diverse cohorts, with longer-term follow-up to assess durability of remission. Integrating advanced imaging modalities such as MRI-based visceral fat quantification could clarify the relative contributions of specific depots to glycemic improvement. Ultimately, consensus definitions of MBS treatment targets, encompassing both metabolic indices, anthropometric and body composition thresholds, may help to standardize reporting, improve patient counselling, and guide clinical decision-making.

## Data Availability

All data supporting the findings of this study are available within the paper and its Supplementary Information.
